# A CFD analysis of equipment fires in an underground development heading for improved auxiliary ventilation design

**DOI:** 10.1007/s42797-025-00119-0

**Published:** 2025-03-26

**Authors:** Oluwafemi B. Salami, Jurgen F. Brune, Guang Xu

**Affiliations:** 1 Department of Mining and Explosives Engineering, Missouri University of Science and Technology, Rolla, MO 65401 USA; 2 Department of Mining Engineering, Colorado School of Mines Golden, Golden, CO 80401 USA

**Keywords:** Underground fire safety, Computational fluid dynamics, Large eddy simulation, Back layering, Critical velocity

## Abstract

**Abstract:**

This study investigates the intricacies of equipment fires in a blind development heading of an underground mine using computational fluid dynamics (CFD). A series of fire dynamic simulations (FDS) were conducted for various ventilation velocities in the main airway, and with different distance between the auxiliary ventilation duct outlet to the blind working face. The impacts of the ventilation velocity in the main airway, and separation distance between the duct outlet to the blind face on temperature distribution and smoke spread mechanism were investigated. The findings indicate that the distance of the auxiliary ventilation duct outlet to the working face has a strong impact on the smoke stratification beneath the airway ceiling. The high-velocity flow from the auxiliary duct leads to turbulent eddies characterized by high levels of fluctuating vorticity near the working face, and the extent of the turbulent region increases as the distance between the working face and the duct outlet increases. This implies that lesser distance between the duct outlet to the working face is safer to mitigate smoke dispersion due to fires in the blind face of an underground heading. Similarly, the ventilation velocity in the main airway was observed to influence the smoke back layering length although, the influence on fire smoke gas temperature in the blind heading was found to be negligible. The insight from this study will aid the future design and installation of auxiliary mine ventilation duct in the underground development heading with the aim of minimizing smoke dispersion and enhancing safe evacuation of personnel in the event of a fire emergency.

****Highlights**:**

- Numerical analysis of a large mining equipment fire is evaluated using CFD
- Auxiliary ventilation duct has a strong impact on fire-induce smoke stratification
- High-velocity flow from auxiliary duct induces turbulent eddies near the blind face
- Turbulent eddies prevent fire smoke stratification which hinders safe evacuation

## Introduction

The blind heading of an underground development face poses a significant risk of fires during key mining activities and accidents in underground mines can lead to catastrophic consequences. In room-and-pillar coal mines for instance, they are the major source of methane and coal dust with a high potential of ignition and explosion (Hansen and Ingason 2013; Feroze and Genc 2017; Hansen 2017; Hansen 2019a, b). Additionally, mobile equipment such as the drilling rig, jumbo drill, continuous miner, etc. engage in long hours of operation in the blind heading of a development face or working face, and thus possess threats of equipment fires which are extremely hazardous to the safety of miners (Conti 2001; De Rosa 2004; Hansen 2009, 2017, 2023; Hansen and Ingason 2013; Hansen 2019a, b; Salami and Xu 2022; Pushparaj et al. 2023). It is noteworthy that, unlike fires occurring mid-tunnel where smoke movement can be influenced by critical ventilation velocities, fires in blind headings restrict smoke flow to a unidirectional path therefore, forcing miners to escape through the smoke-filled tunnel. Since 2000, the United States has experienced over 150 underground mine fires including instances of methane explosions, resulting in severe fatalities (NIOSH 2021). Additionally, numerous reports of face ignitions and spontaneous combustion fires are also reported to the Mine Safety and Health Administration (MSHA) annually.

The upshot of an underground mine fire is primarily voluminous smoke that could be dispersed to other areas of the subsurface environment through the ventilation network which may result into severe fatalities due to the aspiration of CO (Conti 2001; Zhou 2009; Zhou et al. 2018; Bahrami et al. 2021; Salami and Xu 2022; Iqbal et al. 2023; Salami et al. 2023b). Smoke produced from fires in confined spaces such as channels, tunnels, and underground mines accounts for more than 85% of the fatality in confined space environments (Gao et al. 2016; Salami et al. 2023a). The smoke contains noxious gases that could suffocate personnel after descending to a certain height (Long et al. 2022). Furthermore, the smoke reduces visibility thereby seriously hindering emergency evacuation (Salami et al. 2023a, b, 2024). Moreso, the increased implementation of subsurface transportation systems has led to the proliferation of subways and tunnels in urban areas, which in turn has heightened the potential risks associated with fires in these confined spaces (Barbato et al. 2014; Gehandler 2015; Li and Ingason 2018; Haghighat and Luxbacher 2019; Zhang and Huang 2022). Consequently, the dangers of fires in subways and tunnels have become a significant concern in contemporary urban planning and public safety initiatives.

Due to the high cost and difficulty of repeating full-scale tests, numerical approach such CFD have been widely adopted for fire risk assessment. Several researchers have applied CFD to model fire and smoke spread in mines and tunnels (Woodburn and Britter 1996; Hu et al. 2008; Adjiski 2014; Gannouni and Maad 2015; Adjiski et al. 2016; Yuan et al. 2016; Zhu et al. 2016; Fernández-Alaiz et al. 2020), and in the development of management strategies for thermal conditions in underground mines (Sasmito et al. 2015), or for the purpose of evacuation planning (Adjiski et al. 2015). In one study,CFD was used to evaluate the effectiveness of a brattice obstruction to improve safe evacuation and firefighting conditions for underground miners (Adjiski et al. 2016). They analyzed two scenarios of tunnel fires with and without brattice obstruction and the findings showed that using a brattice obstruction could enhance firefighting and safe evacuation of trapped personnel. CFD modeling methods have also been applied to enhance other fire safety measures such as the efficiency of water suppression for conveyor belt fires (Yuan and Smith 2015), carbon monoxide spread during mine fires (Yuan et al. 2016), methane dispersion and methane management in underground mines (Kurnia et al. 2014), and for analyzing the effect of tunnel bifurcation/bifurcation angle on the product of combustion spread in the underground space (Lu et al. 2022a, b; Lu et al. 2022a, b; Salami et al. 2024).

More closely connected to this study is the application of CFD in the development face of an underground heading, and many works have been done to investigate different ventilation design and their efficacy in the underground development heading (Adjiski 2014; Adjiski et al. 2015; Adjiski et al. 2016; Ding et al. 2017; Feroze and Genc 2017; Adjiski and Despodov 2020; Xin et al. 2021; Li, Li et al. 2022). Xin et al. (Xin et al. 2021) employed CFD capabilities to investigate the cooling performance of auxiliary overlap ventilation systems including the far-forcing-near-exhausting (FFNE), and the near-forcing-far-exhausting (NFFE) configurations that are widely used in underground mines. The study found that the NFFE has a superior cooling performance by comparing air velocity, temperature, and relative humidity values in the development heading. Feroze and Genc (Feroze and Genc 2017) utilized CFD to analyze the effect of line brattice ventilation system variables on the airflow near a blind heading. Three factors including the heading dimension, the settings of the line brattice, and the velocity of air from the last through road into the heading were evaluated to estimate the optimum ventilation to the face of the heading. The findings from their study led to the formulation of a user-friendly model that could be used to estimate the flow rate near the face of an empty underground development heading. Another group of researchers led by Li (Li et al. 2022) also applied CFD to determine the appropriate oxygen supply duct type for optimum ventilation strategy in the blind heading of a plateau mine. The results from the study demonstrated that using a slit oxygen outlet has a better oxygen-enrichment effect in the blind heading when compared to traditional oxygen supply method. Likewise, Ding and coauthors (Ding et al. 2017) utilized CFD to examine the airflow distribution in a three-center arch-section tunnel to examine the influence of air velocity and tunnel cross-section on airflow distribution. For the different cases that were examined, they discovered the airflow distribution showed circular pattern, and the average velocity points was observed to be close to the tunnel wall under different airflow velocity.

Despite the enormous literature on underground mine fires, existing understanding of equipment fire dynamics in the development face of underground is very limited, and current studies are insufficient to predict smoke backflow in underground drift due to a fire in the development heading. Classical models on smoke gas temperatures and smoke back layering in previous studies are limited to straight tunnels (Chow et al. 2010; Ingason and Li 2010; Gannouni and Maad 2015; Wu et al. 2018; Haghighat and Luxbacher 2019; Li et al. 2021). In addition, the fire locations are also assumed to be mostly in the main airway of the mine or at the middle of the tunnel (Gannouni and Maad 2015; Wu et al. 2018; Zhao et al. 2018; Li et al. 2021). However, in practical mining situations, most of the equipment fires are likely to occur in the blind heading of the underground airway where the machines are constantly operating. In addition, the smoke spread mechanism would be forced to undergo a unidirectional spread behavior unlike the smoke flow in conventional tunnels. Previous studies have not taken this into account. This study aims to fill this gap by investigating a realistic equipment fire in the blind heading of an underground development heading.

The objective of this study is to investigate the dynamics of an equipment fire in the blind heading of an underground development heading. The key factors including the longitudinal ventilation velocity in the main airway and the distance of ventilation duct outlet to the working face are examined to analyze the fire risk of the equipment fires. A series of fire dynamic simulations with main airway ventilation from 2 to 4 m/s were conducted for different auxiliary duct ventilation setups. This study provides a novel contribution to the existing literature on subsurface fire dynamics and will improve risk assessment framework and emergency preparedness for underground mine fire accidents. The implications of these findings extend to the broader field of fire safety engineering, where the use of CFD modeling has become increasingly prevalent in designing and assessing fire protection systems.

## Model setup

### Lessons learned from experiments

To build a reliable fire dynamic model, lessons learned from previous experiments were incorporated in developing the boundary conditions for this study. In one of the experimental studies earlier conducted, full-scale fire tests were investigated in the underground mine to access the accuracy of FDS (Salami et al. 2024). The environmental and fire field data measured during the full-scale tests were used as the input for FDS simulation and the results showed that FDS is reliable for predicting mine fire evolution. In another similar investigation conducted by Hansen (Hansen and Ingason 2013), mining trucks were set on fire to investigate the heat release rate of burning mining vehicles in the underground mine. The insights drawn from these independent experimental studies have been used to improve the quality and reliability of the findings of this paper.

### Numerical model for this study

**Fig. 1:**
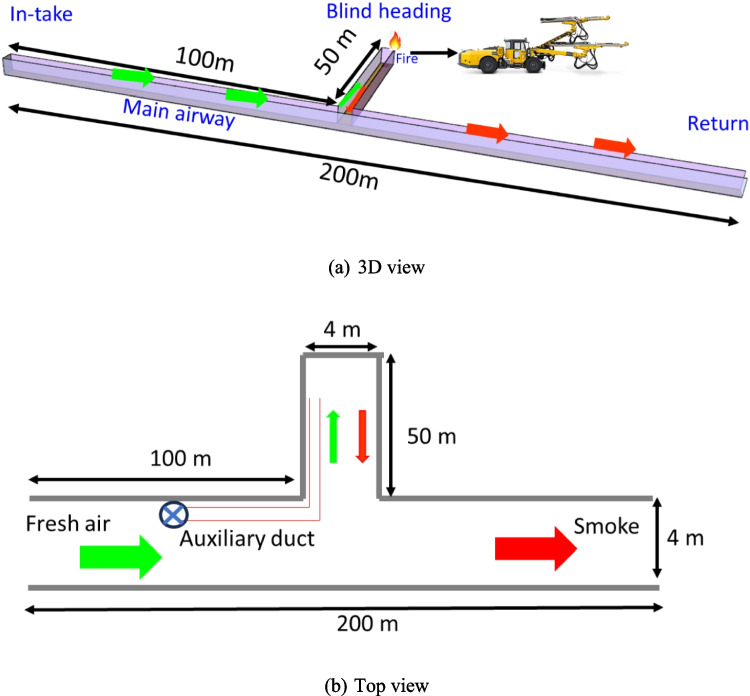
Schematic of numerical model

**Fig. 2:**
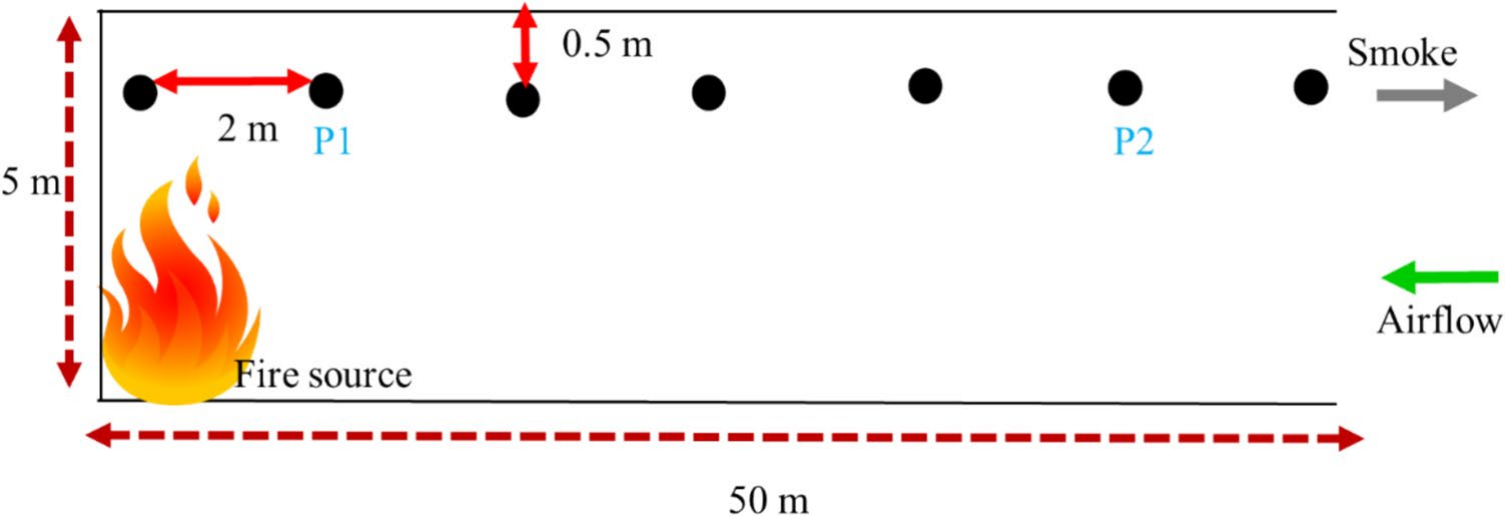
Thermocouples arrangement in the development heading

The fire was assumed to occur during a drilling operation in an underground development face as depicted in Fig. 1. The simulated section of the main airway is 200 m and the development heading is 50 m where the fire occurred. The mine is assumed to be a typical underground development in the US and has a rectangular dimension of width 4 m and height of 5 m. The heat release rate (HRR) value is the most important parameter for fire hazard analysis (Hansen and Ingason 2013; Gannouni and Maad 2015; Hansen 2017; Haghighat and Luxbacher 2019; Salami et al. 2023a). Previous study of a full-scale fire test involving a drilling rig found that the maximum heat release rate of a drilling rig (Atlas Copco Boomer) on fire could be up to 29.4 MW (Hansen and Ingason 2013; Hansen 2017). This value was assumed as the HRR for the equipment fire in this study and the fire source was modeled as a 2 m by 2 m burner in the FDS model. From the equipment specification data sheet, the Atlas Copco Boomer WE3 C has a total power rating of 237 KW (Atlas-Copco 2008). Therefore, by applying the basic ventilation dilution criterion of 0.06 m^3^/s per kw for diesel equipment (Rawlins 2006; Halim 2017), the required airflow to the development was calculated to be 14.2 m^3^/s. This value was used as the input parameter for the flow in the auxiliary ventilation duct to the face. As shown in Fig. 1b, the auxiliary fan is located 5 m upstream of the junction, and the diameter of the duct is 0.6 m. Demonstrated in Fig. 2, FDS thermocouples were installed 0.5 m below the roof of the blind heading and 2 m apart to measure the temperature distribution in the mine during the simulation.

## Numerical simulation

### The solver

One of the most accurate and widely used CFD tools for modeling fire dynamics is the Fire Dynamic Simulator (FDS) developed by National Institute of Standards and Technology (NIST) (Trouvé and Wang 2010; McGrattan et al. 2013a, b; Salami et al. 2023a, 2024). The FDS is an open-source CFD software freely available online (FDS-SMV (nist.gov)). It primarily solve the Navier–Stokes equations for fluid flow with low Mach number (Ma < 0.3)(McGrattan et al. 1998; Gaitonde 2006; Trouvé and Wang 2010; McGrattan et al. 2012; McGrattan et al. 2013a, b; Salami et al. 2023a). FDS can efficiently model low-speed thermally driven flow such as heat and smoke transport phenomenon. Several researchers have applied CFD techniques to solve fire-related problems in mining engineering (Hwang and Edwards 2005; Trouvé and Wang 2010; Cheng et al. 2016; Fernández-Alaiz et al. 2020). Most of the studies demonstrated that FDS generally performs better when compared to other CFD solvers for fire related problems. This is because the solver was primarily designed to solve low-speed thermally driven flow. Additionally, FDS provides more detailed spatial resolutions using less computational time compared to other CFD models (McGrattan et al. 1998; Trouvé and Wang 2010; Salami et al. 2023a).

### FDS simulation

In this study, the effect of two key factors on temperature distribution and smoke backflow were examined. They include intake airway velocity, and the separation distance between the ventilation duct to the blind heading. The intake airway velocities of 2 m/s, 3 m/s, and 4 m/s were investigated while the ventilation duct outlet was placed 10 m, 15 m, and 20 m from the blind heading. A total of nine simulations were conducted. The detailed numerical simulation cases are presented in Table 1.

**Table 1:** Simulation cases for this study

Distance of auxiliary fan development from blind heading (m)	Intake airway velocity (m/s)	Simulation case [Distance (m), velocity (m/s)]
10	2	[10, 2]
	3	[10, 3]
	4	[10, 4]
15	2	[15, 2]
	3	[15, 3]
	4	[15, 4]
20	2	[20, 2]
	3	[20, 3]
	4	[20, 4]

### Simulation parameters and boundary conditions

In this study, the default setting for dynamic turbulence modeling was retained for the simulation setup. This default settings adopt the constant Smagorinsky model (where ${P}_{rt}=0.5,$ ${S}_{ct}=0.5,$ $and {C}_{s}$=0.17). The measured HRR from the experiment field tests (Hansen and Ingason 2013; Hansen 2020) was set as the heat release rate per unit area (HRRPUA) in the FDS simulations. The reaction type was set as “HEPTANE’’, and the “SOOT_YIELD = 0.1”. The exhaust of the modelled geometry was modeled as “OPEN” surface because of it connects to ambient environment (See (McGrattan and Forney 2000) for further information on setting boundary conditions in FDS). Similarly, the intake of the modeled geometry was modeled as “SUPPLY”. The different airflow rates were assigned to the supply based on the set simulation scenario. The mine walls were set as “CONCRETE” and the surfaces of the wall were assigned “INERT” for the FDS simulation. This concrete option for mine wall property has been successfully used by previous studies for mine fire modeling with FDS (Wu et al. 2020; Peng et al. 2022; Salami et al. 2024; Yao et al. 2023; Zhu et al. 2023),. The wall and ceiling material possessed a density of 2100 kg/m^3^, specific heat of 879 J/(kg K), and thermal conductivity of 1.10 W/(m K) (Seike et al. 2017), while the thickness of the wall was set to 0.2 m. However, because FDS treats the wall as a thin obstruction which results in a computational error when the mesh size is increased from 0.2 m to 0.5 m for sensitivity studies, “Thicken” was applied to the obstruction properties of the wall for the FDS simulation of mesh sizes 0.4 m and 0.5 m to prevent simulation error. Additionally, to ensure that the numerical results are reliable and computationally correct, the time step is constrained such that the Courant-Friedrichs-Lewy (CFL) condition is satisfied (Cheong et al. 2009; Gannouni and Maad 2015):

1 $$\delta t max \left(\frac{u}{\delta x},\frac{v}{\delta y},\frac{w}{\delta z}\right)\le 1$$

The initial time step is specified automatically in FDS by dividing the grid size by the characteristic velocity of the flow. The value of the time step is given as

2 $$\frac{5{(\delta x\delta y\delta z)}^{1/3}}{\surd gH}$$

**Fig. 3:**
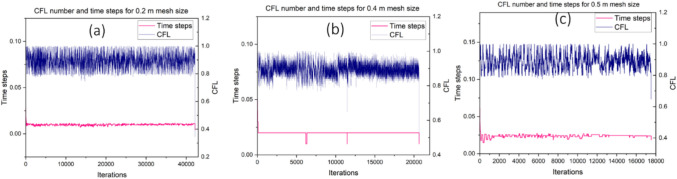
CFL number and time steps for different mesh sizes (**a**) 0.2 m mesh size (**b**) 0.4 m mesh size (**c**) 0.5 m mesh size

For each time step during the calculations, the velocities $u$, $v$, $w$ are tested to ensure that the CFL condition is met. The CFL number and time steps obtained for different mesh sizes during the simulation are presented in Fig. 3(a−c) The CFL numbers are between 0.8 to 1.0 for all the cases. All three cases satisfy the stability criterion and indicate that the model is computationally correct.

The fire was simulated using a high-performance laboratory computer with the latest version of FDS, FDS 6.7.7 at the time of this simulation. The main airway and the development heading domain were divided into four continuous meshes each and the entire mine domain has eight meshes. Each mesh was assigned to an MPI, and the number of Open MP processes was set to 2. This will enable the calculations in each computational mesh to be done in parallel to speed up the processing time. The ambient temperature inside and outside the mine was set to the default value of 20 ^o^ C in the FDS while the pressure was set to 101325.0 Pa just like in the previous experimental studies (Salami et al. 2024). The simulation time was set to 420.0 s (which implies that the drilling rig fire was assumed to burn ten times faster compared to the real scale experimental time of 70.0 min (4200.0 s). The reason for this is to reduce the computational resources of simulating for days since the major fire evolution could be captured within the current simulation framework andLarge eddy simulation (LES) was adopted as the turbulent model.

### Turbulence model

LES adopts the closure model used in describing unresolved convective transport for fire in confined spaces (Trouvé and Wang 2010). The steps given below explain how to set up the LES simulation for fire in a confined space such as an underground mine (Rodi et al. 1997, McGrattan 2005, McGrattan et al. 2013a, b):

First, an evolution equation is formulated for the kinetic energy of the gas by taking the dot product of the momentum equation and the velocity vector ***u***:

3 $$\rho \frac{D{\varvec{u}}}{Dt} . {\varvec{u}}\boldsymbol{ }\equiv \rho \frac{D\left(\frac{{\left|{\varvec{u}}\right|}^{2}}{2}\right)}{Dt}=\dots - \varnothing$$

The sink term, $\varnothing$, known as the dissipation function can be obtained from the viscous stress tensor $\tau$, and the velocity vector as given by Eq. (4).

4 $$\varnothing =\tau .\nabla u=\mu \lfloor 2{\left(\frac{\partial u}{\partial x}\right)}^{2}+\dots \rfloor$$

The sink term also shows up in the energy equation with the extra terms hidden for the sake of clarity and simplicity. The expression is merely explaining in mathematical terms how the kinetic energy of the flow is converted into thermal energy due to viscosity.

5 $$\frac{D}{Dt}\left(\rho h\right)=\dots + \varnothing$$

The dissipative effect of the viscosity can thus be represented as a large-scale flow simulation by the expression:

6 $$\mu_{LES}=\rho\left(C_s\Delta\right)^2{\lfloor(2\left(\frac{\delta\overline u}{\delta x}\right)^2+\dots\rfloor}^{1/2}$$

where:

${C}_{s}$ is an empirical constant generally taken to be equal to 0.2 (Rahmani et al. 2004),

$\Delta$ is the grid size of the cell, and the term in bracket has the same functional form as the dissipation function.

The thermal conductivity and material diffusivity are related to the LES viscosity by:

7 $${k}_{LES}\boldsymbol{ }=\frac{{\mu }_{LES}{c}_{p}}{{P}_{r}} ; {D}_{LEs}=\frac{{\mu }_{LES}}{{\rho S}_{c}}$$

where ${P}_{r}$, is the turbulent Prandtl number and ${S}_{c}$ is the Schmidt number.

### Mesh sensitivity study

The grid size is the principal factor that determines the resolution of the CFD simulation and could impact simulation results. For this reason, appropriate grid sensitivity should be done to obtain mesh independence. In FDS, the proper grid size can be derived by the fire characteristic diameter given in Eq. (8) (McGrattan et al. 2013a, b; Weng et al. 2015):

8 $${D}^{*}={\left(\frac{\dot{Q}}{{\rho }_{\infty }{c}_{p}{T}_{\infty }\sqrt{g}}\right)}^{2/5}$$

where $\delta x$ denotes the nominal size of the mesh cell, $\dot{Q}$ represents the total heat release rate of the fire (kW), ${\rho }_{\infty }$ designates the ambient air density kg/m^3^, C_p_ is the specific heat capacity of air (KJ/kg/k), ${T}_{\infty }$ is the ambient temperature (K), and $g$ is the acceleration due to gravity (usually taken as 9.81 m/s^2^) (McGrattan et al. 2013a, b; Overholt 2014; McGrattan et al. 2016).

The ratio of fire characteristic size to grid size (${D}^{*}/\delta x$) known as the plume resolution (PR) index is normally used to describe the quality of the calculation grid (Gannouni and Maad 2015). The higher this value is, the finer the meshes are and the more computational time is required for the CFD simulation. However, sensitivity studies from the literature have recommended that values between 4 to 16 are sufficient to obtain an appropriate resolution with minimal computational requirements (McGrattan et al. 2013a, b; McGrattan et al. 2014). The mesh size for this simulation is also determined by this rule. For this study, the computational domain is obtained by setting the$x$, ${x}{\prime},$ $y,{y}{\prime}, z$, and ${z}{\prime}$ to −1.0, 201.0, −1.0, 5.0, −1.0, 6.0 for the main underground drift and 99.0, 105.0, 5.0, 55.0, −1.0, 6.0 for the development face crosscut. Here, where$x$,$y$, $z$ represents the minimum values and${x}{\prime}$, ${y}{\prime}, {z}{\prime}$ represents the maximum values for the coordinates $x$, $y$, $z$ respectively.

**Fig. 4:**
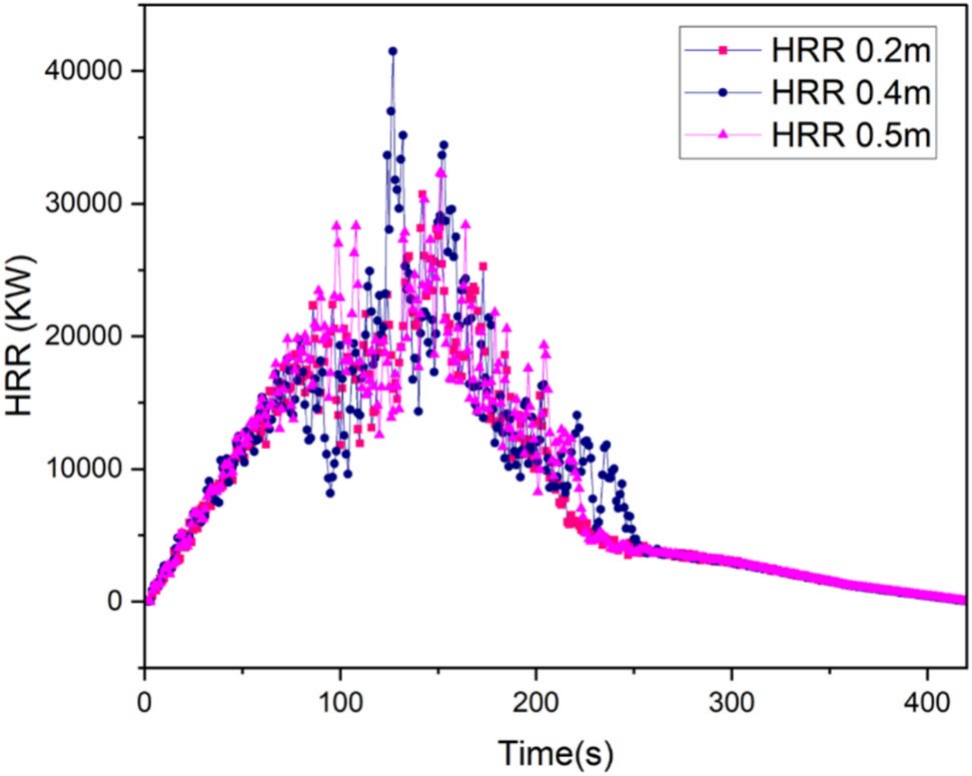
Comparison HRR time history for different mesh sizes

**Fig. 5:**
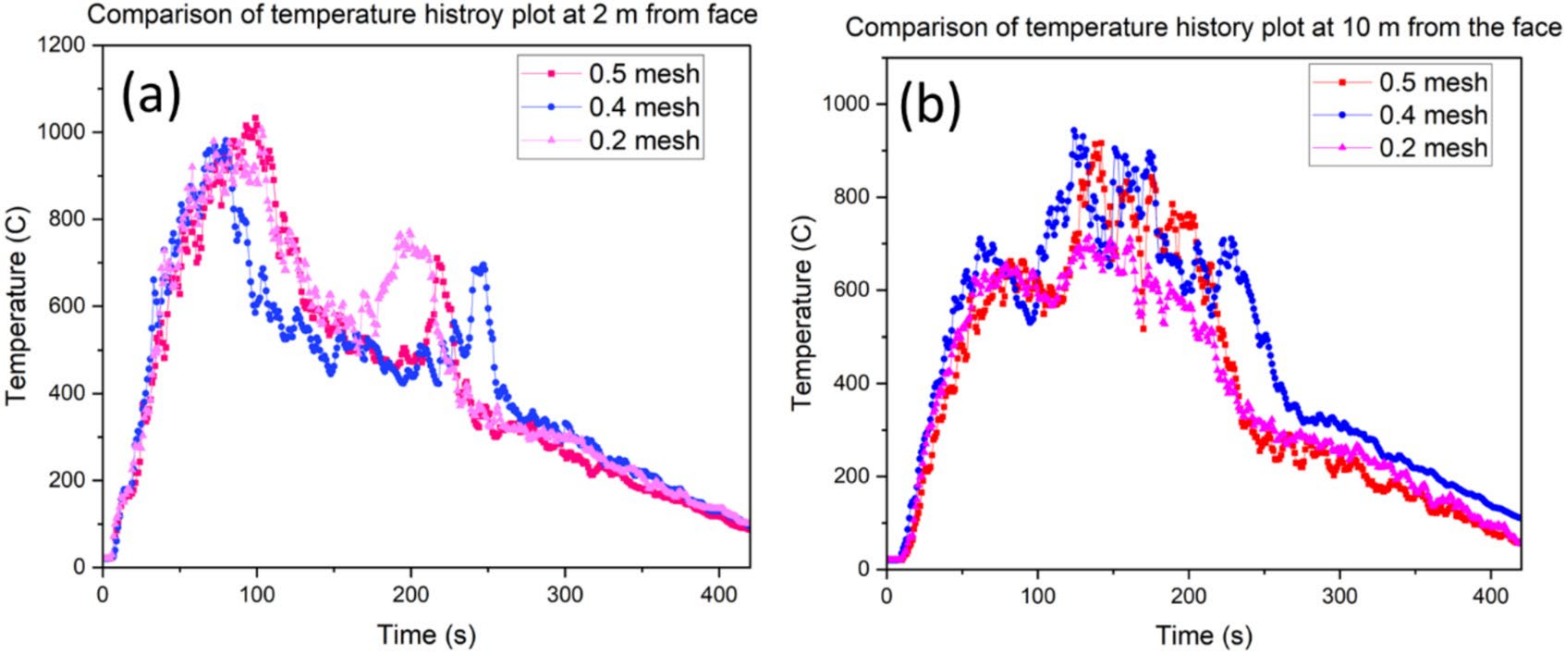
Comparison of temperature history plots at stations P1 and P2 along the crosscut (**a**) At 2 m from face (**b**) At 10 m from face

According to Hasen, the calculated maximum HRR for the drilling rig is 29.4 MW (Hansen 2017; Hansen 2019a, b). The mesh sensitivity study is conducted based on this HRR value by using different mesh sizes to determine the suitable mesh for the desired accuracy before further computation. The characteristics fire diameter ${D}^{*}$ computed is 3.67. From the calculated fire characteristics diameter, difference mesh sizes are computed as presented in Table 2. A comparison of the HRR and temperature history plots is presented in Figs. 4 and 5 respectively. As seen in Fig. 5, the temperature measured at points P1 and P2 (see Fig. 2) shows that the mesh sizes have very close history plots and that reducing the mesh size does not significantly influence the temperature value, however, it could significantly increase the computational resources. Station P1 and P2 are 2 m and 10 m from the blind heading respectively. By comparing Fig. 5a and b, the values in Fig. 5b match better compared to Fig. 5a. This may be due to the high turbulence near the fire and usually, studies have shown that smaller mesh sizes around the fire zone could improve the temporal resolution of the computational domain. Hence, a mesh size of 0.4 m was chosen as adequate for this study and applied for subsequent calculations. The summary of the mesh parameter is presented in Tables 2 and 3.

**Table 2:** Computed mesh size for sensitivity studies based on ${D}^{*}$

Mesh type	Ratio	Computed mesh size (m)
Coarse	${D}^{*}/5$	~ 0.7
Medium	${D}^{*}/10$	~ 0.4
Fine	${D}^{*}/16$	~ 0.2

**Table 3:** Mesh parameters

Case scenario	Mesh size Δx × Δy × Δz	The total number of cells in the model	Simulation time (hr.)
Model 1	0.2 × 0.2 × 0.2	1,323,000	32.84
Model 2	0.4 × 0.4 × 0.4	170,100	7.39
Model 3	0.5 × 0.5 × 0.5	84,672	3.71

### Model validation

**Fig. 6:**
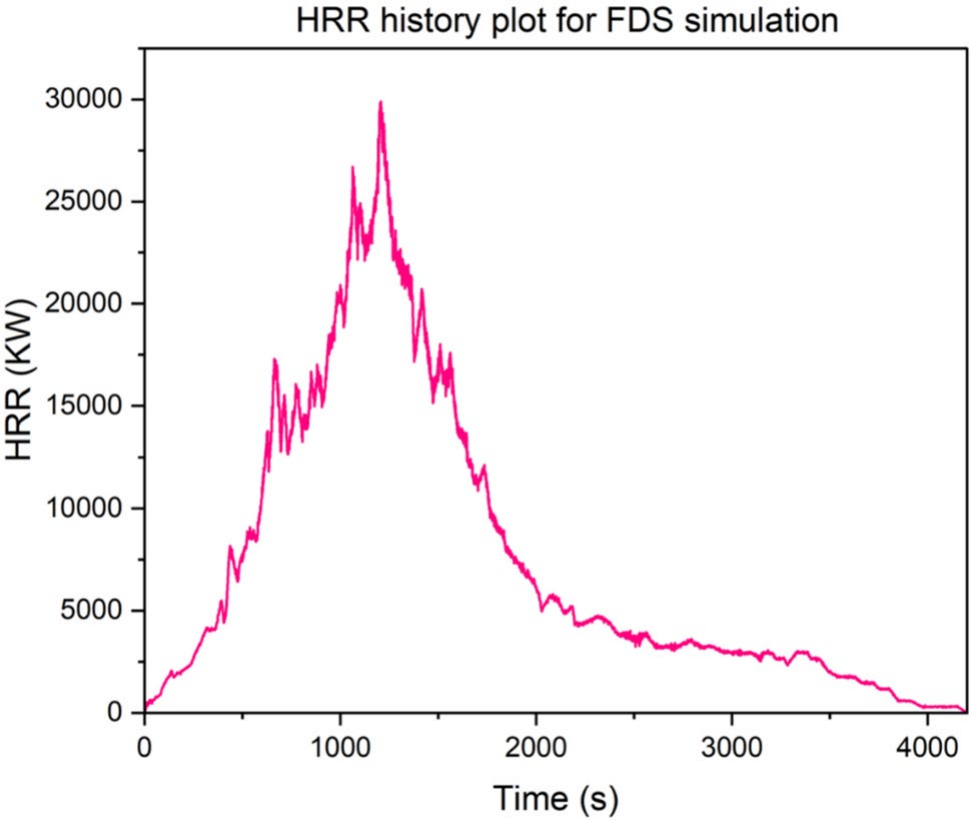
HRR time history plot from Hansen full-scale experiment (Hansen and Ingason 2013; Hansen 2017, 2020)

Model validation is important to ensure credibility and reliability. To ensure credibility of these results, an independent model was built for the purpose of validation. The model was developed using the experimental scenario reported by Hansen involving a mining equipment (drilling rig) fire tests (Hansen and Ingason 2013; Hansen 2017, 2020). The measured HRR from the experiment is depicted in Fig. 6. This HRR history plot was used as the input for the simulation to mimic the experimental condition by invoking a “Fire_RAMP” function in the FDS code.

The simulation boundary conditions were set based on the experimental conditions (See Appendix for FDS validation code). The drift dimension was roughly 6 m by 8 m. The location in the drift where the experiment took place was approximately 100 m from the inlet. The drilling rig was located at approximately 54 m from the exhaust drift. The exhaust drift was approximately 40 m. Before the fire experiment, a tempest fan was placed at the beginning of the drift (approximately 46 m from the drilling rig) to push the smoke to the exhaust. The fan’s capacity was 217 000 m^3^/hr., and this was modeled as a “SUPPLY” inlet with a volume flow rate of 60.27 m^3^/s for the FDS simulation. All other properties such as reaction type, wall properties, and HRR are set as described in Section "Simulation parameters and boundary conditions".

**Fig. 7:**
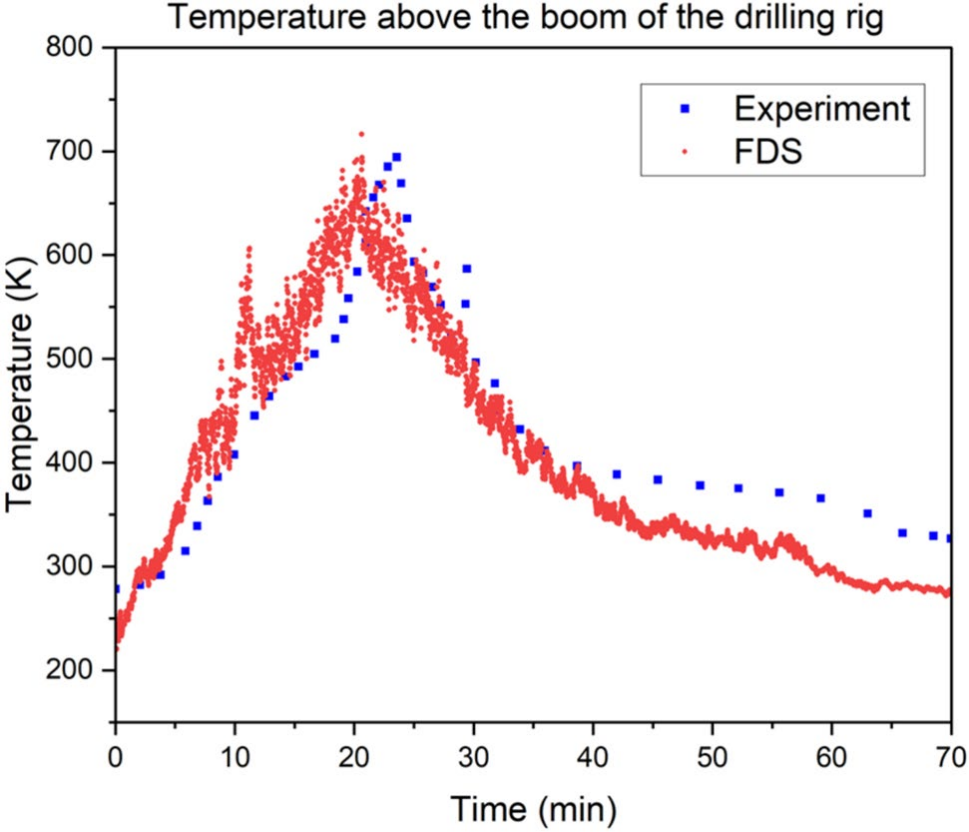
Comparison of fire gas temperature at thermocouple Tc35 for the drilling rig fire test

During the experiment, a thermocouple was installed at the top of the boom of the drilling rig to measure the temperature. The length of the Rocket Boomer drilling rig with boom was 12.4 m. In the simulation setup, a thermocouple was installed at 12 m from the center of the fire to depict the boom of the drilling rig. A comparison between the experimental values and the predicted value from the simulation is presented in Fig. 7. The results from the CFD modeling were found to fit the experimental data very well. Most importantly, the model developed in this study predicted the maximum ceiling temperature to a high degree of accuracy which indicates that the model is reliable. A comparison of the model prediction performance with similar studies reported in literature by Yuan et al.’s model (Yuan and Smith 2015; Yuan et al. 2016), Fernandez et al. (Fernández-Alaiz et al. 2020), and Hansen (Hansen 2020) also indicated that this model validation is acceptable by a mere visual comparison of the experimental and predicted data trends.

**Fig. 8:**
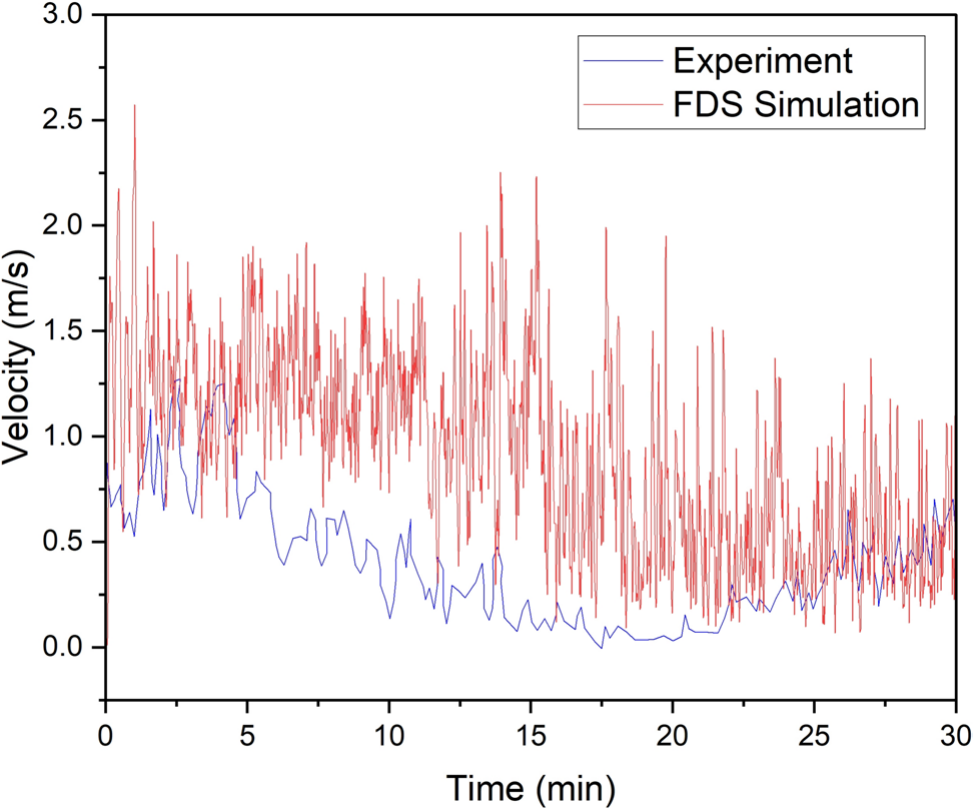
Comparison of velocity 4.4 m below the ceiling at the middle of the exhaust

Similarly, a comparison of the velocity probe measurement that was installed approximately 4.4 m below the roof at the exhaust drift is presented in Fig. 8. The result of the CFD modeling was found to fit the experimental values very well during the initial and extinction phase of the fire although, it overestimated the fire gas velocities during the combustion phase. This has been earlier reported by Hansen (Hansen 2020), and the increased differences was observed to coincide with the initiation of a higher fire growth rate and higher heat release rates during the drilling rig experiment which the CFD model does not seem to properly account for.

## Results and discussion

### Effect of intake airway velocity

**Fig. 9:**
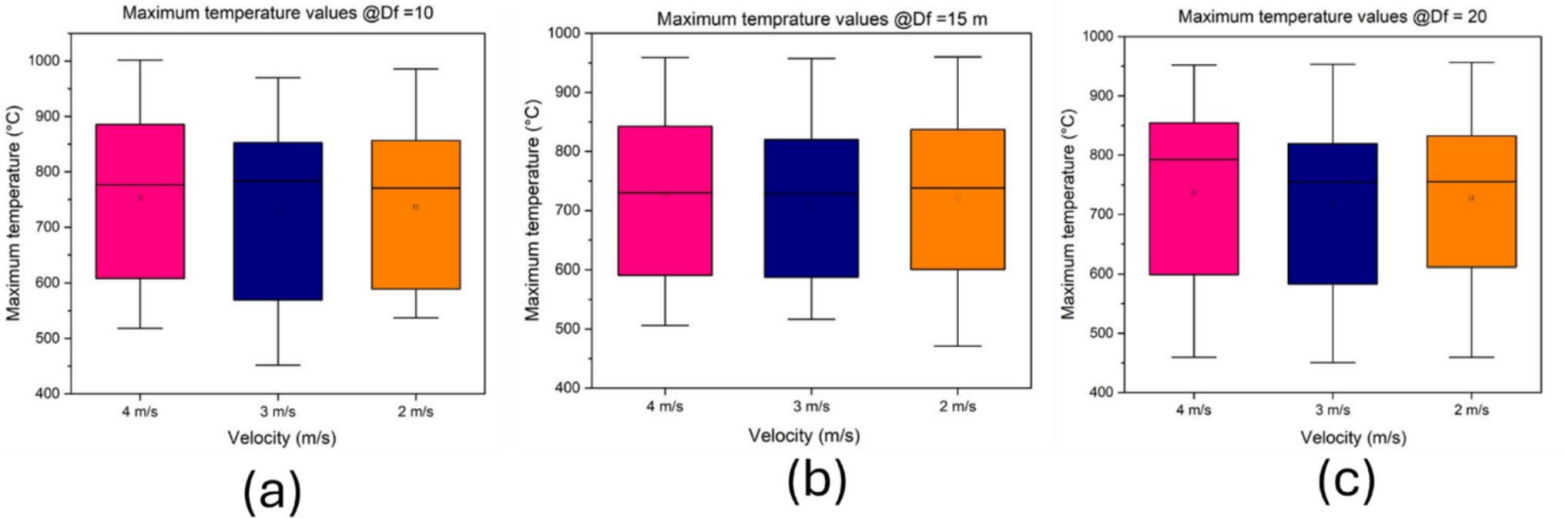
Temperature variance @${D}_{f}$ =10 m, 20 m, and 30 m from blind face (**a**) 10 m from face (**b**) 15 m from face (**c**) 20 m from face

Figure 9a-c shows the maximum ceiling temperature distribution along the development heading for various intake airway velocities. The maximum, minimum, and mean temperature values for the different ventilation conditions are very similar and the intake airway ventilation in the main airway does not have a significant impact on the measured temperature values beneath the ceiling of the development heading.

In Fig. 9a, the maximum and minimum temperature was approximately 1000 °C and 500 °C respectively for intake velocities of 4 m/s and 2 m/s. However, there was a slight difference in the simulated maximum and minimum temperature for velocity of 3 m/s which may be due to turbulence fluctuations, and this is not very significant. In Fig. 9b and Fig. 9(c), the maximum and minimum temperatures for the different velocities are approximately 950 ^o^ C and 500 ^o^ C. The temperature values are relatively the same for the same value of${D}_{f}$. However, the maximum temperature decreases slightly as ${D}_{f}$ is increased. Although previous studies have shown that ventilation have an impact on temperature in straight tunnels, this study examines the impact on temperature along a development heading which mainly depends on auxiliary ventilation rather than longitudinal ventilation.

### Effect of ${D}_{f}$ on temperature stratification

**Fig. 10:**
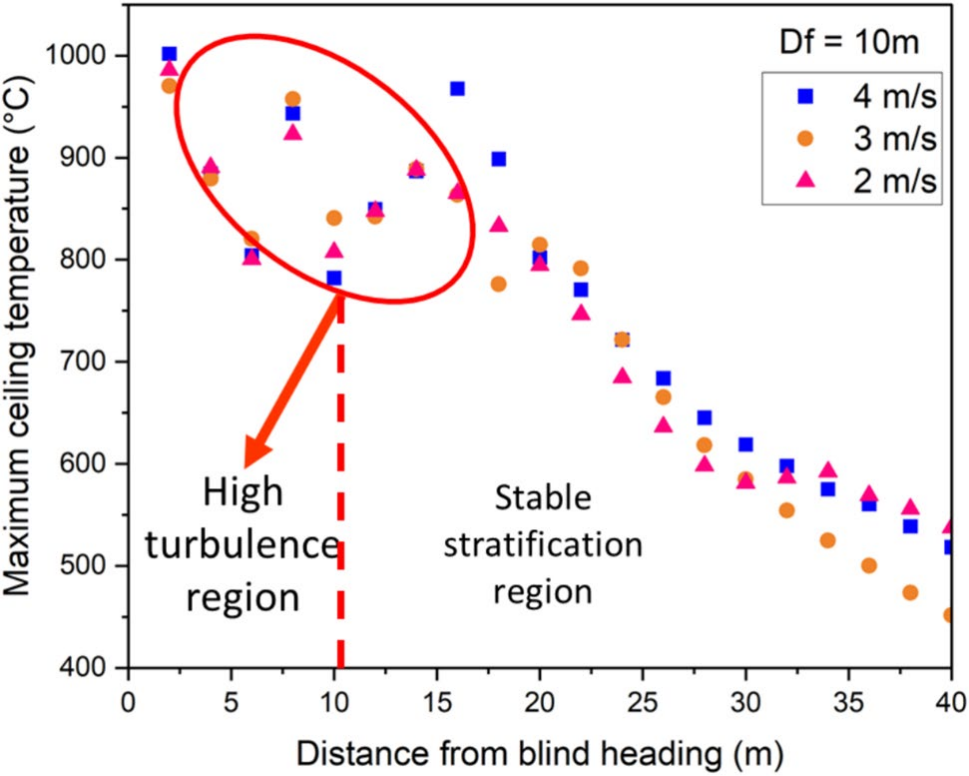
Maximum ceiling temperature for different longitudinal velocities at ${D}_{f}$ =10 m

**Fig. 11:**
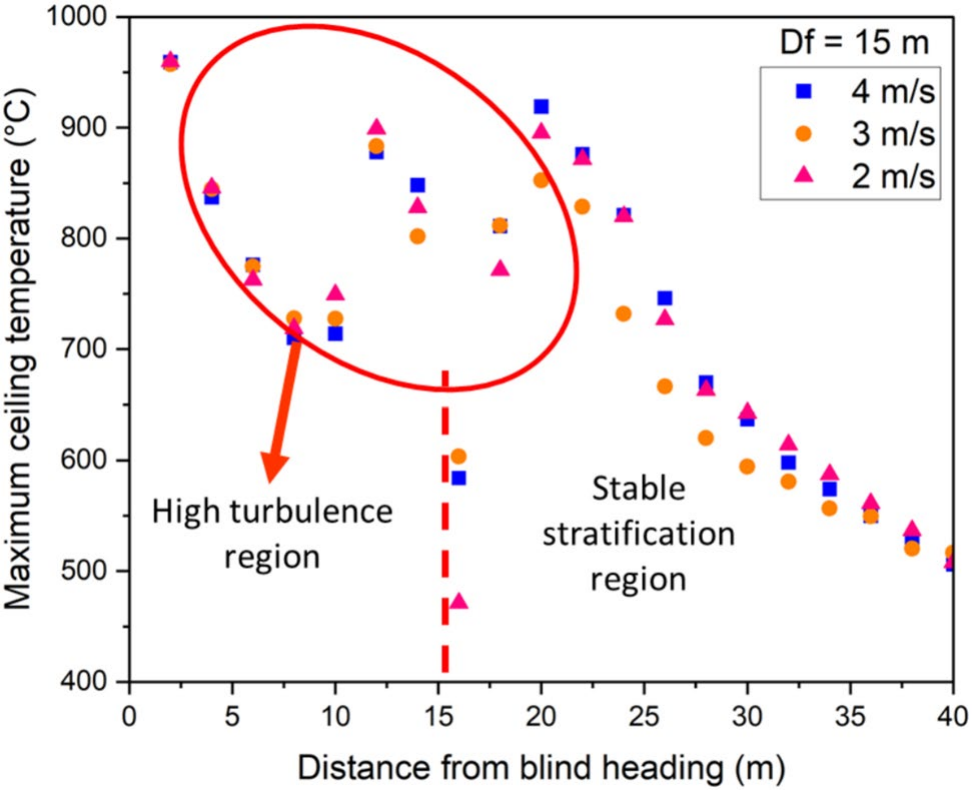
Maximum ceiling temperature for different longitudinal velocities at ${D}_{f}$ =15 m

**Fig. 12:**
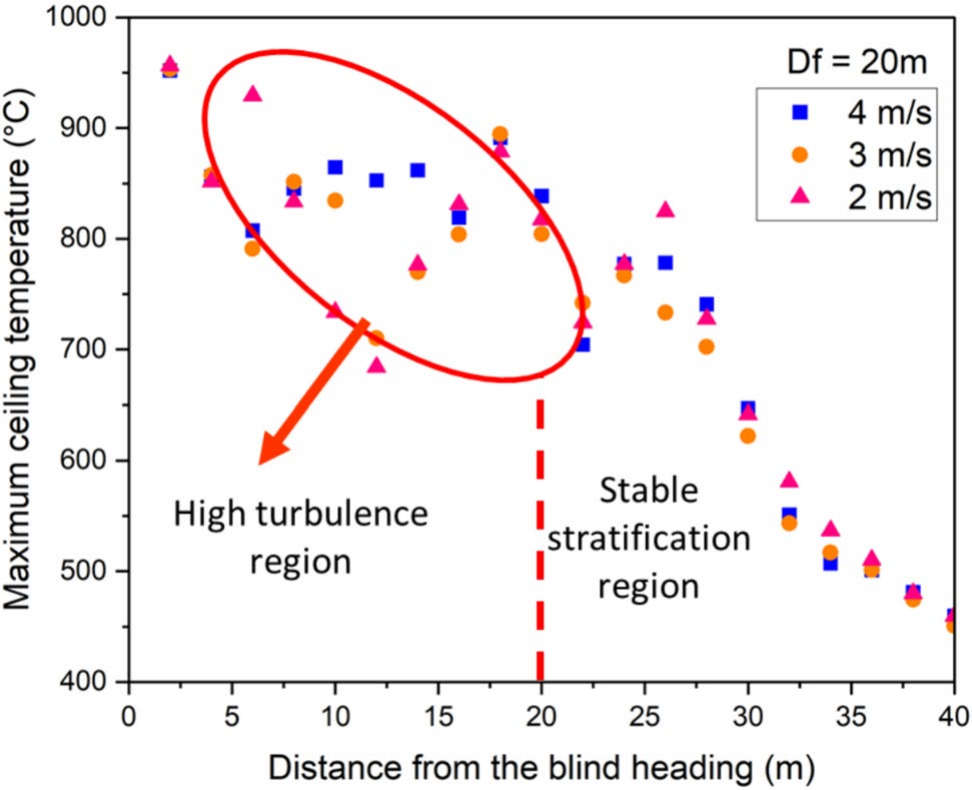
Maximum ceiling temperature for different longitudinal velocities at ${D}_{f}$ = 20 m

Figures 10, 11, and 12 depicts that the position of the auxiliary ventilation duct has a strong impact on the temperature stratification beneath the roof of the development heading. For instance, in Fig. 10, the presence of the ventilation duct divides the development heading into two regions: (1) a region of high turbulence and, (2) a region of stable stratification. A high turbulence region is observed between the face and the location of duct outlet. It can be observed that there is poor smoke layer stratification from the outlet of the ventilation duct to the development face due to the high-speed flow from the ventilation duct. The high-velocity flow opposes the natural upward movement of the fire smoke. This disruption could lead to the fire smoke becoming unstable, causing fluctuations in temperature (as depicted in the region of high turbulence from Figs. 10, 11, and 12).

Similarly, in Figs. 11 and 12, the high flow velocity creates turbulence in the airflow beneath the blind heading. The interaction between the high-velocity flow and the fire smoke leads to turbulent eddies characterized by high levels of fluctuating vorticity. Beyond the auxiliary ventilation outlet, there is a relatively stable layer of smoke along the development heading ceiling. A comparison between Figs. 10, 11, and 12 indicates that the extent of the turbulent region increases as the distance between the blind face and the auxiliary ventilation duct increases. When the distance between the auxiliary ventilation duct and the blind is 10 m, the turbulent region was observed to be approximately 14 m from the face. This increased to roughly19 m when ${D}_{f}=15 m$, and to approximately 24 m when ${D}_{f}$ was increased to 20 m.

### Velocity and smoke backflow

**Fig. 13:**
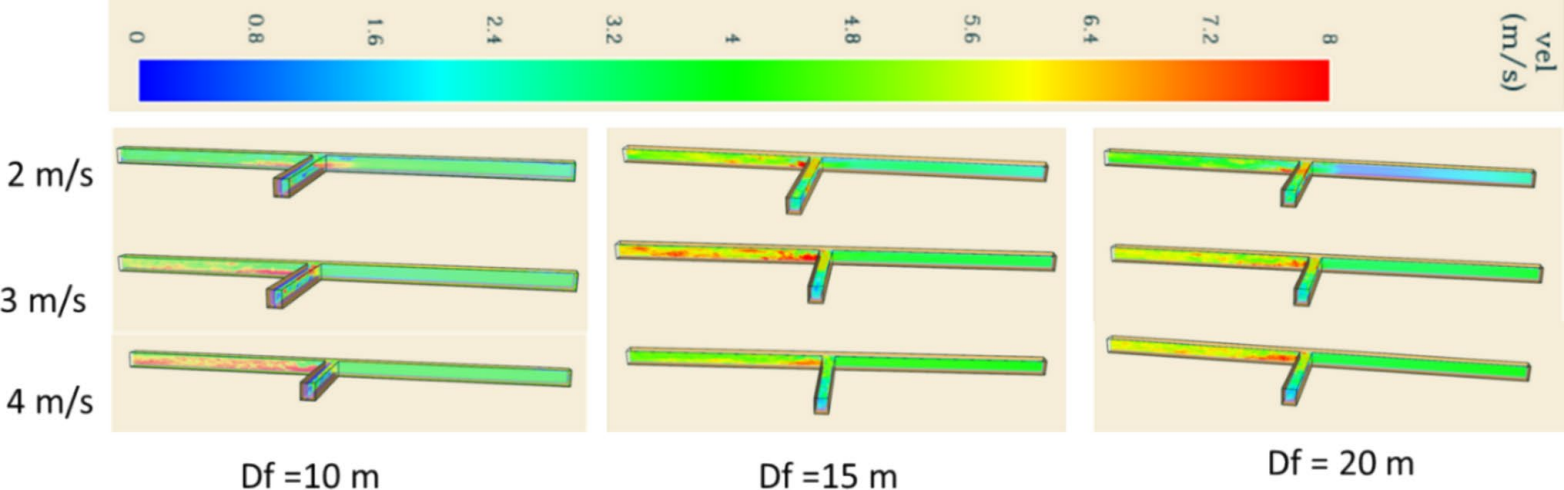
Velocity at 160 s

The velocity profile measured at 160 s during the fire simulation is presented in Fig. 13. The time was selected because the smoke backflow was observed to be maximum at this time. The velocity in the smoke region downstream of the development heading has higher average values due to fire smoke mix which leads to complex interaction between different air layers. The distance of the auxiliary ventilation does not impact the airflow in the development heading and the main airway.

**Fig. 14:**
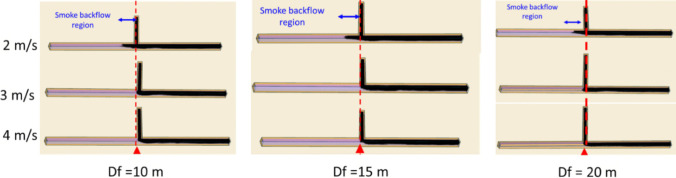
Smoke backflow at 160 s

**Fig. 15:**
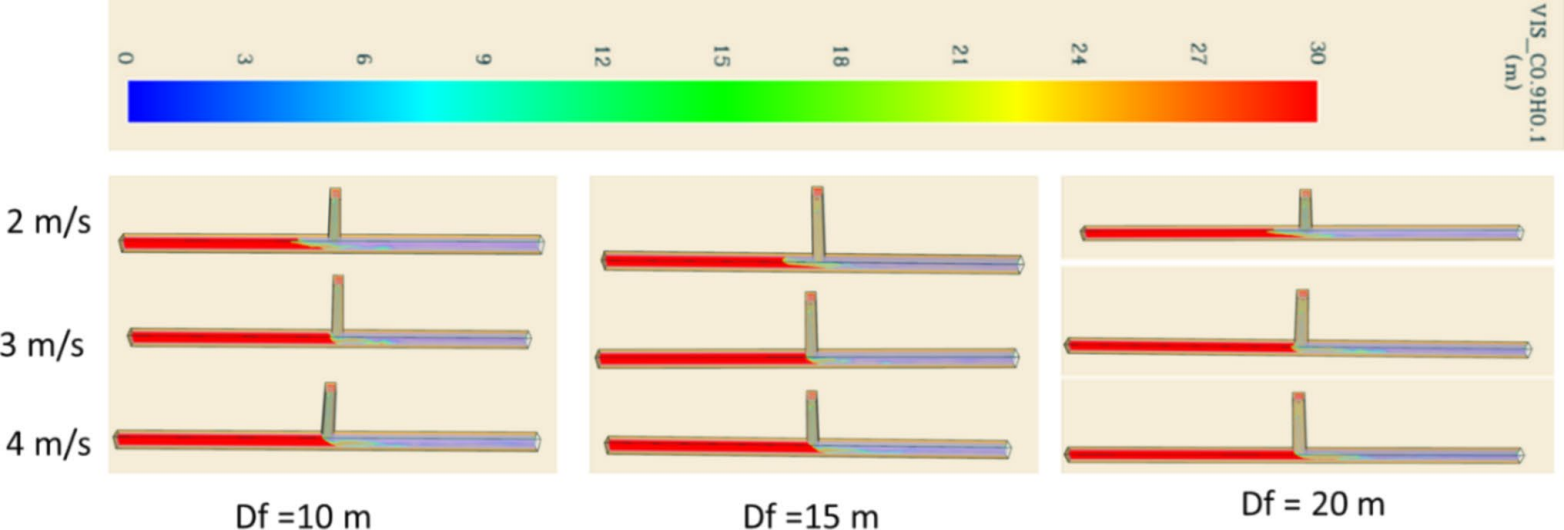
Visibility at 160 s

Figure 14 illustrates the smoke dispersion patterns under different scenarios. Notably, it shows the substantial impact of the intake airway ventilation velocity on the smoke reversal occurring in the main airway after smoke comes out from the development heading. Specifically, at a ventilation velocity of 2 m/s, the smoke exhibited a backflow phenomenon from the blind heading junction, extending approximately 15 m. Conversely, when the ventilation rate was increased to 3 m/s, the entirety of the smoke was effectively directed toward the exhaust. The impact of smoke spread on visibility condition is presented in Fig. 15. It shows that the visibility is directly impacted by the fire smoke. The region filled with smoke tends to have poor visibility compared to the smoke-free region. The visibility was observed to reduce from 30 m to approximately 5 m during the peak of the fire in the blind heading and towards the exhaust.

**Fig. 16:**
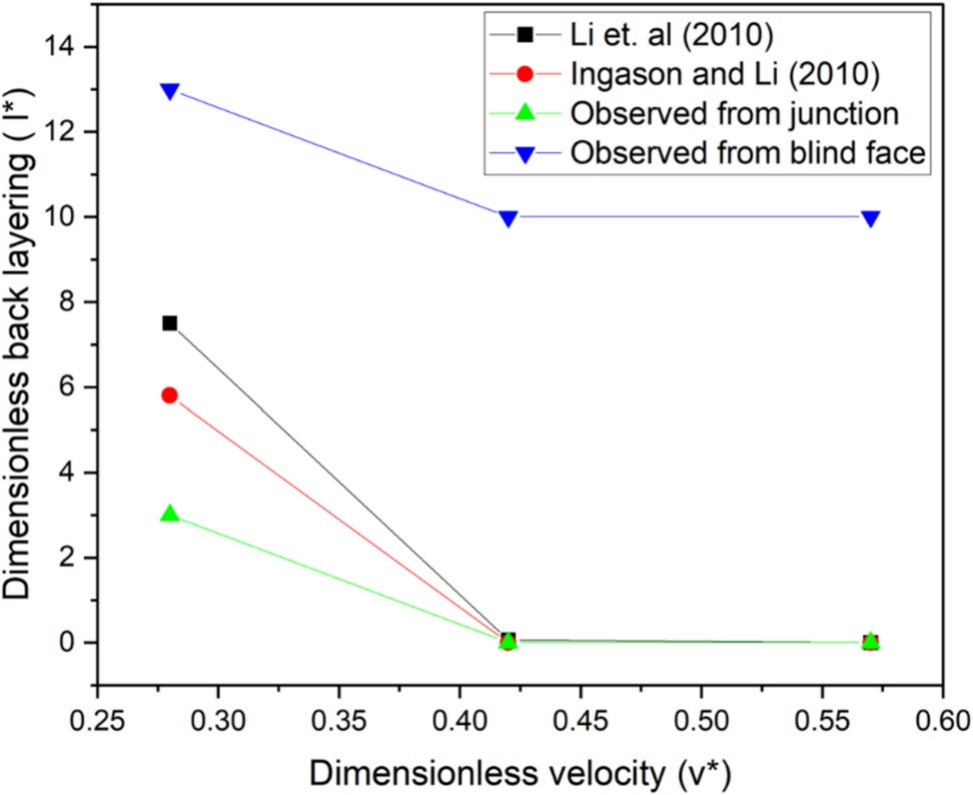
Dimensionless back layering vs dimensionless velocity

The result from this investigation indicates that existing empirical model for predicting back layering in straight tunnels do not account for the influence of bifurcation such a fire in a blind heading. For instance, Fig. 16 shows that even though the calculated back layering length and critical ventilation velocity observed from the blind heading junction could be predicted to a reasonable accuracy by exiting empirical models, exiting empirical model such as (Ingason and Li 2010) and (Li et al. 2010) underpredicted the back layering length when the total smoke backflow length from the blind face was taken into account. This implies that existing back layering models are mostly suitable for fires in straight tunnels and further work needs to be done to incorporate the influence of bifurcation of smoke back layering length. This was not investigated in this study and would be examined in subsequent research.

## Conclusions

In this study, numerical investigations of underground fire events due to an equipment fire in the blind heading was investigated. A numerical model was developed, and the model was validated using the notable mining equipment fire experiment conducted by Hansen. The measured temperature for the model shows good agreement with the observed values during the full-scale experiment. The maximum temperature beneath the airway of the development heading was analyzed for various distances between the outlet of the auxiliary ventilation duct and the blind face, and under different ventilation conditions. The findings indicated that the distance of the auxiliary ventilation duct from the development face has a strong impact on the fire smoke stratification beneath the ceiling of the main airways. The fire smoke gets disrupted due to the high-speed flow from the duct outlet, this leads to increased energy dissipation as a result of the turbulence generated. This effect becomes more pronounced as the distance of separation between the blind face and the ventilation duct is increased. As this distance increases, the turbulence region of fire smoke increases, leading to increased dispersion of fire smoke. Previous studies have shown that smoke reduces visibility and could impede the safe evacuation of personnel. This study suggests that ventilation duct outlet position could prevent smoke stratification and thus hinder the safe and timely evacuation of occupants trapped in an underground development heading. Additionally, smoke backflow in the main airway was investigated and the critical ventilation velocity was found to be approximately 3.0 m/s for this scenario. This value will be much greater if the fire location was changed from the blind face and placed in the middle of the tunnel. This corroborates the argument from this paper that fire in a blind heading presents a unique fire dynamics compared to fires in straight tunnels and future work will involve series of experimental studies to investigate this phenomenon.

## Data Availability

No datasets were generated or analysed during the current study.
